# Indications and use of therapeutic phlebotomy in polycythemia vera: which role for erythrocytapheresis?

**DOI:** 10.1038/s41375-018-0304-9

**Published:** 2018-12-05

**Authors:** Luciana Teofili, Caterina Giovanna Valentini, Elena Rossi, Valerio De Stefano

**Affiliations:** Fondazione Policlinico Universitario A. Gemelli IRCCS and Istituto di Ematologia, Università Cattolica, Roma, Italy

**Keywords:** Therapeutics, Quality of life

**Fig. 1:**
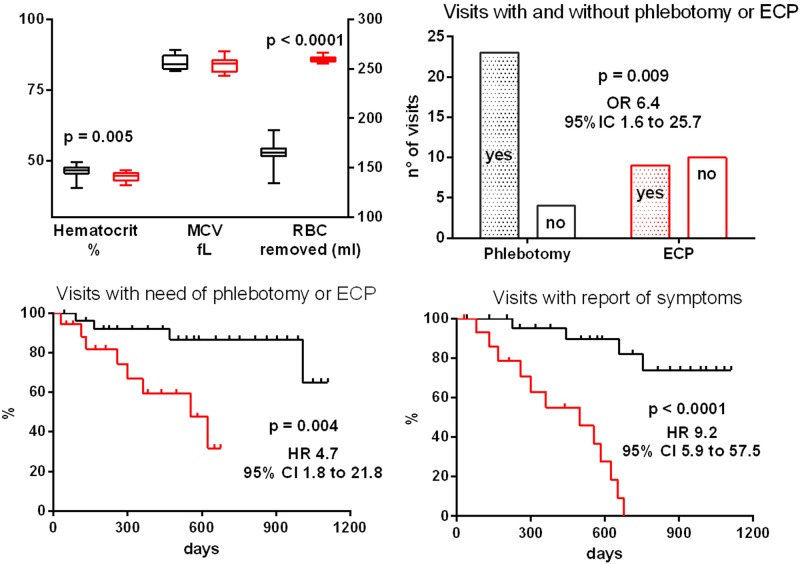
Laboratory and clinical findings during phlebotomy (black) and ECP (red) therapy periods

The Italian Societies of Hematology and Blood Transfusion issued recent recommendations for phlebotomy in polycythemia vera (PV), to obtain a target hematocrit <45% [1]. Selective red blood cell (RBC) apheresis (erythrocytapheresis, ECP) is recommended as an alternative to phlebotomy only when a rapid attainment of the target hematocrit is needed, such as occurrence of severe vascular complications, or before emergency surgery [1]. We suggest an additional indication, offering ECP to those patients who require numerous rounds of phlebotomy, and have contraindications or unwillingness to use cytoreductive therapy. Here we report an emblematic case of a PV patient diagnosed at the age of 39 years; her main symptom was severe acromelalgia. From 2001 to 2016, she received low-dose aspirin and a median number of 7 phlebotomies/year (range 5–8), on average every 47 days (range 22–124). Over this time, she recurrently expressed her discomfort with the high phlebotomy requirement, but she was extremely concerned to start interferon or hydroxycarbamide. In September 2016, ECP was implemented in an attempt to reduce the phlebotomy rate. Figure 1 compares the findings of the ECP period (September 2016–August 2018) to those of the previous 3 years with phlebotomies (September 2013–August 2016). No signs of myelofibrotic evolution were present. The median RBCs removed were 165 ml (range 134–188) after phlebotomies and 259 ml (range 256–266) after ECP. ECP resulted in a straighter hematocrit control; the frequency of procedures gradually lowered, with a median interval between ECP of 58 days (range 28–152), and acromelalgia completely disappeared (Fig. 1). No adverse reaction occurred. Overall, this case suggests that ECP may be attempted if phlebotomy fails to control hematocrit and/or PV-related symptoms.

Isovolemic ECP removes a large RBC volume without affecting donor blood volume, tailoring the withdrawal to both pre-procedure and target hematocrit. However, the use in PV patients has been scarcely explored [2–4]. ECP and phlebotomy were employed in 30 and 99 PV patients, respectively, during a period of 3.5 years. In comparison with phlebotomies, ECP removed more RBC volume and lowered hematocrit better, requiring half-procedures [4]. The interval between procedures is reported to be longer after ECP: 20 days–4 months after phlebotomy and 4–7 months after ECP [2–4]. The major efficacy of ECP over phlebotomy has been confirmed in two randomized trials in patients with hemochromatosis [5, 6].

The main concerns against the routine management of PV patients by ECP are apheresis-related adverse events and the high cost [1]. In two series of 62 and 40 patients with erythrocytosis, the rate of ECP-related adverse events was <2% [3] and up to 32.5% [4], respectively. All events were attributable to the hypocalcemia caused by citrate in the ACD-A anticoagulant, but were mild and without need of calcium supplementation [4]. Indeed, mild citrate-induced symptoms (perioral tingling, malaise, nausea, and chills) occur in up to 80% of healthy apheresis donors. Severe symptoms (convulsions and laryngeal spasm) occur up to 0.4% of procedures [7], with a rate comparable with the 0.1–0.5% rate of severe adverse vasovagal reactions recorded during whole blood donations [7].

ECP is about 3.5-fold more expensive than phlebotomy, either due to the higher cost of devices or because of the indirect costs due to the longer time employed by specialized personnel [3, 5, 6]; the difference in the total costs is only partially mitigated by the longer interval after ECP [5, 6]. However, among hemochromatosis patients, ECP results in less hours of absence from work and less costs of lost production, with an overall cost per procedure lower by one-third in comparison with phlebotomy [5].

Up to 25% of PV patients perceive phlebotomies as having a negative impact on quality of life (QOL) and productivity, and up to 8% of patients discontinue phlebotomies because they feel worse after treatment, or for the inconvenient frequency of visits [8]; in this regard, lowering the frequency of procedures, likewise maintaining a control of hematocrit and of symptoms, is an important clinical need.

A randomized trial in PV patients managed by RBC withdrawal could be appropriate, investigating the different effects of ECP and phlebotomy on target hematocrit, frequency of procedures, disease-associated symptoms, vascular complications, working activity, and QOL, as well as iron deprivation and its clinical consequences [9]. The cost–efficacy analysis of ECP should consider all these outcomes.
